# Bta-miR-665 improves bovine blastocyst development through its influence on microtubule dynamics and apoptosis

**DOI:** 10.3389/fgene.2024.1437695

**Published:** 2024-10-16

**Authors:** Xuefeng Guan, Yuan Fan, Rani Six, Camilla Benedetti, Annelies Raes, Andrea Fernandez Montoro, Xiaole Cui, Nima Azari Dolatabad, Ann Van Soom, Krishna Chaitanya Pavani, Luc Peelman

**Affiliations:** ^1^ Department of Veterinary and Biosciences, Faculty of Veterinary Medicine, Ghent University, Merelbeke, Belgium; ^2^ Department of Internal Medicine, Reproduction and Population Health, Faculty of Veterinary Medicine, University of Ghent, Merelbeke, Belgium; ^3^ Department for Reproductive Medicine, Ghent University Hospital, Gent, Belgium

**Keywords:** MicroRNAs, blastocyst formation, embryo development, microtubules, cell proliferation

## Abstract

Extracellular vesicles (EVs) contain microRNAs (miRNAs), which are important regulators of embryonic development. Nevertheless, little is known about the precise molecular processes controlling blastocyst development and quality. In a previous study, we identified bta-miR-665 as one of the miRNAs more abundantly present in extracellular vesicles of embryo-conditioned culture media of blastocysts compared to degenerate ones. Here, we investigated the effect and regulatory roles of bta-miR-665 in blastocyst development by supplementation of bta-miR-665 mimics or inhibitors to the culture media. Supplementation of bta-miR-665 mimics improved cleavage and blastocyst rate (*P* < 0.01), and blastocyst quality as indicated by increased inner cell mass rates and reduced apoptotic cell ratios (*P* < 0.01). Furthermore, supplementation of bta-miR-665 inhibitors had the opposite effect on these phenotypes. Low input transcriptome analysis and RT-qPCR revealed that bta-miR-665 acts on genes linked to microtubule formation and apoptosis/cell proliferation. These insights not only elucidate the important role of bta-miR-665 in embryo development, but also underscore its potential in improving reproductive efficiency in bovine embryo culture.

## Introduction

While more and more roles of microRNAs (miRNAs) are unveiled, their impact on embryonic development still remains largely unknown (Seidel, 2015). With the current artificial reproduction technologies, such as *in vitro* maturation (IVM), *in vitro* fertilization (IVF) and *in vitro* culture (IVC), the majority of bovine oocytes can fully mature and start to cleave during *in vitro* culture. However, only about 30% of the fertilized zygotes develop into blastocysts and only half of the blastocysts successfully implant after transfer (Pavani et al., 2020). In contrast, the natural development of embryos *in vivo* shows an implantation rate of 65%, indicating that bovine blastocysts produced *in vitro* are of inferior quality (Demetrio et al., 2020). Many miRNAs, along with other signaling factors (such as proteins, transcription factors and metabolites, have been found to influence embryo development and some of them can be used to predict the quality of blastocysts (Mutia et al., 2023; Zhou and Dimitriadis, 2020; Fang et al., 2021a; Kropp and Khatib, 2015). Nevertheless, the underlying mechanisms of how they affect blastocyst formation and quality remain largely unknown.

MicroRNAs are a type of endogenous RNA with a length of 22 nucleotides that regulate gene expression post-transcriptionally (Ambros, 2004; Lu and Rothenberg, 2018). They can be transferred within the organism in various ways, for example, bound to lipoproteins or enclosed in extracellular vesicles (EVs) (Machtinger et al., 2016), and act as intercellular communicators. Furthermore, miRNAs are highly stable, especially the ones contained in vesicles, and they can be detected in different biofluids, including serum, plasma, saliva, urine, semen, amniotic fluid, and milk (Salilew-Wondim et al., 2020; Weber et al., 2010). In previous studies, we identified miR-378 present in the embryonic culture medium as an indicator of pre-implantation developmental competence through its effect on hatching and reduction of apoptosis (Pavani et al., 2022) and miR-30c, miR-10b and miR-146b were identified as indicators of developmental arrest since they reduce DNA repair and increase apoptosis in embryos (Lin et al., 2019a; Lin et al., 2019b; Pavani et al., 2023). Studies about microRNA expression in bovine somatic cell nuclear transfer embryos and bovine preimplantation embryos has shown that miRNAs are involved in elimination of maternal messenger RNAs (mRNAs) and promote embryonic genome activation (Berg and Pfeffer, 2018; Cuthbert et al., 2021). Fluctuations in miRNA levels ensure that the intricate processes of embryo development are executed with the required temporal and spatial precision, allowing cells to respond appropriately to environmental signals (van Wijk et al., 2022). MiRNAs can help to maintain the balance between stem cell pluripotency and lineage commitment, thus ensuring the proper formation of tissues and organs (Yao, 2016). Additionally, they also allow adjustments in cellular conditions, safeguarding the developing embryo against potential disruptions (Gross et al., 2017) and are therefore vital for normal progression of embryonic development (Paulson et al., 2022).

Cell-free circulating microRNAs are more susceptible to RNase degradation compared to those encapsulated in EVs (Kim et al., 2017; Coenen-Stass et al., 2019), thus microRNAs in EVs may offer more specificity and stability, making them more reliable markers for pre-implantation embryo development. We previously identified 69 differentially expressed microRNAs in EVs secreted by degenerated embryos compared to blastocysts. Among them, bta-miR-665 was more abundant in EVs secreted by blastocysts, suggesting that a certain level of bta-miR-665 is needed for early development of bovine embryos (Pavani et al., 2022).

The aim of this study was to determine whether bta-miR-665 has a positive effect *in vitro* embryonic development and to gain insight in the targeted molecular mechanisms. In this way, we demonstrated that bta-miR-665 positively impacts the development and quality of bovine embryos and alters the expression of genes related to apoptosis and embryonic microtubule dynamics.

## Materials and methods

### Media and reagents

Tissue culture medium (TCM)-199, minimal essential medium (MEM), non-essential amino acids (100×), synthetic basal medium eagle amino acids, gentamycin, and kanamycin were purchased from Life Technologies Europe (Ghent, Belgium). Phosphate-Buffered Saline (PBS) was obtained from Gibco™ (Catalog number: 2001–2019, Thermo Fisher Scientific, Waltham, MA, United States). All other chemicals were obtained from Sigma-Aldrich (Diegem, Belgium). All media were filtered before use (0.22 μM Pall Corporation, Ann Arbor, MI, United States).

### Oocyte preparation and *in vitro* maturation (IVM)

Cow ovaries were obtained at the local slaughterhouse and processed within 2 h after collection. Upon arrival at the lab, the ovaries were washed three times in warm physiological saline (with 25 mg/mL kanamycin). Cumulus-oocyte complexes (COCs) were aspirated from antral follicles, sizing between 2–8 mm diameter, using an 18-gauge needle attached to a 10 mL syringe. Subsequently, viable oocytes with uniformly granulated cytoplasm and surrounded by more than three compact layers of cumulus cells were selected and cultured in 500 mL modified bicarbonate buffered TCM-199 (supplemented with 50 mg/mL gentamicin and 20 ng/mL epidermal growth factor) in 5% CO_2_ in air for 22 h at 38.5°C.

### *In vitro* fertilization (IVF)

Frozen-thawed bull spermatozoa were separated by using a 45/90% Percoll^®^ gradient (GE Healthcare Biosciences, Uppsala, Sweden) centrifugation. The sperm pellet was washed in IVF–Tyrode’s albumin–pyruvate–lactate (TALP) medium, containing bicarbonate-buffered Tyrode solution. The sperm concentration was adjusted to a final concentration of 2 × 10^6^ spermatozoa/mL using IVF–TALP medium enriched with BSA (Sigma A8806; 6 mg/mL) and heparin (25 mg/mL). After 22 h of maturation, bovine oocytes were washed in 500 µL IVF-TALP and subsequently co-incubated with bull spermatozoa. After 21 h gamete co-incubation, presumed zygotes were vortexed to remove surplus zona attached cumulus and sperm cells.

### *In vitro* culture (IVC) and treatments

As previously demonstrated by our group, bovine serum albumin (BSA) (Sigma A9647) is not a possible source of foreign miRNA contamination (Pavani et al., 2022; Pavani et al., 2018). Therefore, 4 mg/mL BSA (Sigma A9647) was supplemented to synthetic oviductal fluid enriched with non-essential and essential amino acids synthetic oviductal fluid (SOF) and ITS (5 μg/mL insulin; 5 μg/mL transferrin; 5 ng/mL selenium). The presumed zygotes were culture in 50 µL droplets (control) covered with Paraffin oil (SAGE™ oil for tissue culture, ART-4008-5P, a Cooper Surgical Company, Målov, Denmark), at 38°C in 5% CO_2_, 5% O_2_ and 90% N_2_. The bta-miR-665 mimics were obtained from Sangon (Shanghai, China) and added in SOF medium of group cultured zygotes at a final concentration of 2 μM in 50 µL medium droplet. Treatment groups were set up as mimics (adding bta-miR-665 mimics), inhibitor (adding bta-miR-665 inhibitor), mimics negative control (NC, SOF with the addition of a scrambled oligonucleotide with no homology to the bovine genome), inhibitor control (inNC, SOF with the addition of an inactive analog of the inhibitor), Control (=SOF medium without any other addition) (Supplementary Figure S2) (25 zygotes per replicate, Supplementary Table S4). In total, grouped droplets of 2,456 presumed zygotes were cultured. At 45 h post insemination (hpi), embryo cleavage was scored as the percentage of cleaved embryos out of presumed zygotes. At 8 dpi, the developmental competence of each embryo was assessed, enabling a division of embryos into two subgroups: (1) embryos that were not able to reach the blastocyst stage (degenerated embryos) and (2) blastocysts. Nine replicates of each group were gathered for further analysis. Three replicates with 25 blastocysts for miRNA RT-qPCR analysis and three replicates of 3 × 35 = 105 blastocysts for transcriptomics. The treatment details are shown in Supplementary Figures S1, S2, and the embryos growth state at Day 8 is presented in Supplementary Figure S3.

### Differential staining

The blastocysts were washed three times in PBS (containing 0.5% BSA) at 37°C for 2 min each time and then fixed in 2% paraformaldehyde for half an hour. The blastocysts were then put into permeabilization solution (PBS solution containing 0.5% TritonX-100% and 0.05% Tween-20, 20µL/blastocyst) for 30 min at room temperature. Then the permeabilized blastocysts were washed three times with PBS (0.5% BSA) at room temperature for 2 min each time. Blastocysts were treated in 1 mol/L HCL solution for 30 min at room temperature and then moved into 100 mM/L Tris-HCl (PH = 8.4) for 20 min at room temperature. After treatment, blastocysts were washed 3 times in 0.5% BSA-PBS at room temperature for 5 min each time. Then, the blastocysts were transferred to blocking solution (PBS containing 10% goat serum stacking and 0.05% Tween-20) and blocked overnight at 4°C. The blastocysts were placed in the primary antibody (CDX2 and Caspase3) diluted in 1:500 diluted blocking solution and incubated overnight at 4°C. After primary antibody incubation, blastocysts were washed three times with 0.5% BSA-PBS for 15 min each time. Then blastocysts were put into the secondary antibody dilution in 1:500 blocking solution and incubated at room temperature for 2 h in the dark. Subsequently, blastocysts were washed three times in 0.5% BSA-PBS and dyed (including Negative Control) in 20 μM/L Hoechst-33342 for 10 min at room temperature, then washed three times in 0.5 BSA-PBS. 5 μL of anti-quenching agent DABCO was put on a glass slide and 1-3 blastocysts were placed in each droplet. The blastocysts were observed under a fluorescence microscope and photographed using a confocal microscope (Supplementary Figures S3, S4).

### RNA sample library construction and sequencing

Five groups of blastocysts were collected at 8 dpi (control, NC mimic, NC inhibitor, bta-miR-665 mimic, bta-miR-665 inhibitor). For each group, total RNA was isolated from three biological repeats of 3 × 35 = 105 blastocysts using the RNeasy Micro kit (Qiagen, Germantown, United States) according to the manufacturer’s protocol and then pooled per group (Takele Assefa et al., 2020). The quality and concentration of the RNA samples were examined using an RNA 6000 Pico Chip (Agilent Technologies, Carlsbad, CA, United States) and a Quant-iT RiboGreen RNA Assay kit (Life Technologies, Carlsbad, CA, United States), respectively. Transcriptome library preparation was done by BGI, the trimmed reads were mapped against the *Bos taurus* genome (GCF_002263795.1_ARS-UCD1.2). Differential expression analysis between the groups of samples (Control, NC mimic, NC inhibitor, bta-miR-665 mimic, bta-miR-665 inhibitor) was performed with EdgeR (Love et al., 2014). And differential gene expression analysis was performed using PossionDis on Dr. Tom platform (SOP-SS-053 BGI). This resulted in a list of enriched pathways and differentially expressed genes that are part of those pathways (Tang et al., 2023).

### Total RNA extraction and reverse transcription

On average, 25 blastocysts from each group were collected, washed by PBS, then stored at −80°C and used for RNA extraction. RNA extraction was performed using the Qiagen miRNeasy Micro Kit. Three replicates of 25 blastocysts of each treatment group were used. A Nanodrop 1000 Spectrophotometer (Themo Fisher Scientific, Wilmington, DE, United States) was used to quantify total RNA. Next, cDNA was synthesized using the LNA First Line Start Kit (Qiagen, German) according to the manufacturer’s protocols. For detection of bta-miR-665 and the small nuclear RNA U6 control, total RNA was extracted from culture medium (100 μL), degenerated embryos (n = 25) and blastocysts (n = 25) as previously mentioned. Reverse transcription (RT) reactions were performed in 20 µL mixtures containing 2 μL of total RNA, 1 μL of U6 Spike-in (5 μM), 2 μL 10x Enzyme Buffer mix, 4 μL 5x dNTP Premix buffer, 11 μL ddH_2_O. RT was performed as follows: 42°C 60 min, 95°C 5 min, stored at −20°C for no more than 6 months for future use.

### Gene expression analysis by RT-qPCR

Reverse transcription quantitative real-time polymerase chain reaction (RT-qPCR) was performed on blastocysts, degenerated embryos, and culture medium from each group in 20 μL mixtures containing: 2 μL primers (0.1 nM/μL), 2 μL sample cDNA (50 ng), 5 μL SYBR Green Premix Buffer, 1 μL ddH_2_O. Amplification and gene expression levels were measured using a real-time thermal cycler, CFX Connect™ Real Time System (BIO-RAD, CA, United States). We used a three steps cycling protocol: denaturation at 95°C for 15 min, denaturation for 15 s, annealing at the selected temperature for 30 s and extension at 72°C for 30 s for 50 cycles, and reading after every cycle (Supplementary Table S1). The stably expressed *GAPDH* was used for normalization. All reactions were performed in triplicate, and the 2^−ΔΔCt^ method was used to analyze the data (Livak and Schmittgen, 2001). Abbreviation of genes are shown in Supplementary Table S2.

### Statistical analysis

Manually collected data (RT-qPCR; primers; differential staining) were exported to Microsoft Excel (Microsoft Corp., Redmond, WA), where data exploration and organization were done using the PivotTables function (Microsoft Excel). All experiments were done in at least three replicates and analyzed using GraphPad PRISMs ver.5.01 (PRISM 5; GraphPad Software, Inc., San Diego, CA, United States). Generalized mixed-effects models were used to test the effects of miRNA mimics and inhibitor supplementation (Control, mimics Negative Control, inhibitor Negative control, bta-miR-665 mimics, bta-miR-665 inhibitor) on developmental rates (cleavage and blastocyst ratio) expressed as a percentage from presumed zygotes. RT-qPCR data was analyzed using univariate analysis of variance (ANOVA) followed by Tukey’s test. Differences were considered statistically significant at *P < 0.05, **P < 0.01, ***P < 0.001.

## Results

### Higher bta-miR-665 levels in blastocysts compared to degenerated embryos

**FIGURE 1:**
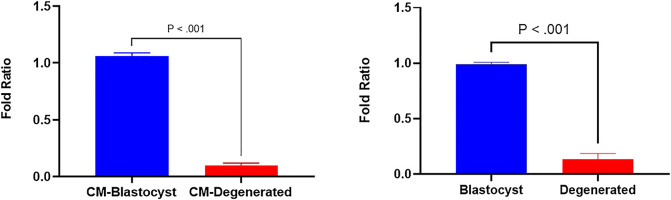
Differential expression of bta-miR-665 in blastocysts and degenerated embryos and their culture media. Results are expressed as mean ± SE.

In a previous study, we identified several differentially expressed (DE) miRNAs in EVs derived from the conditioned media of blastocysts and degenerated-embryos (GEO accession number: GSE197878) (Pavani et al., 2022). The sequencing data revealed significantly higher levels of bta-miR-665 in the EVs derived from blastocyst conditioned media compared to that of degenerated embryos. To verify this result, we performed RT-qPCR to detect the bta-miR-665 expression levels in both embryos and embryo derived culture media. RT-qPCR showed bta-miR-665 was indeed significantly enriched in blastocysts and their culture media compared to the degenerative ones (Figure 1, *P* < 0.001).

### Bta-miR-665 positively influences blastocyst formation and embryo quality

**FIGURE 2:**
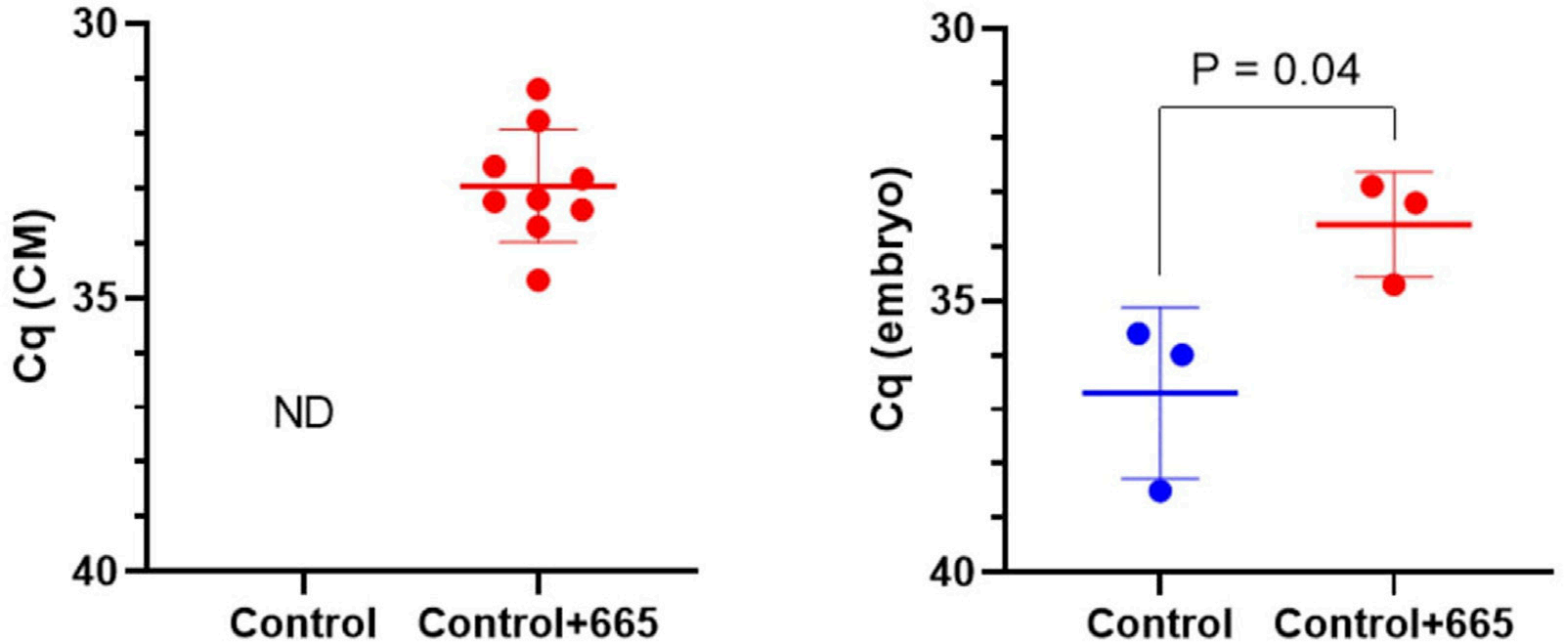
MiR-665 levels in media and zygotes after supplementation of miR-665 mimics were determined by using RT-qPCR. Results are expressed as least-square mean ± SE. (ND: not detected).

To investigate the functional effects of bta-miR-665 on embryo development, we supplemented bta-miR-665 mimics or inhibitors into the *in vitro* culture medium of presumed zygotes. RT-qPCR indicated a significant increase of bta-miR-665 levels in embryos after supplementation of bta-miR-665 mimics to the culture medium compared with the control group cultured in synthetic oviductal fluid (SOF) (Control group) at Day 2. This result demonstrates that bta-miR-665 mimics supplemented to the culture medium are taken up by embryos (Figure 2).

Adding bta-miR-665 mimics (2 μM in 50 μL) to the culture medium (n = 482, 19 replicates) increased the blastocyst ratio by 8.37% ± 2.09% (vs. mimics Negative Control, n = 477, 19 replicates), 13.49% ± 1.26% (vs. inhibitor-665, n = 475, 19 replicates), 8.72% ± 2.23% (vs. inhibitor Negative Control, n = 479, 19 replicates), 6.95% ± 1.04% (vs. Control, n = 543, 22 replicates) (Table 1). Adding bta-miR-665 inhibitor to the culture medium (n = 475) decreased the blastocyst ratio by 4.77% ± 1.17% (vs. inhibitor Negative Control, n = 479), 6.54% ± 0.21% (vs. Control, n = 543) (*P* < 0.05) (Table 1).

**TABLE 1:** Cleavage (2 dpi) and blastocyst (8 dpi) rate of group cultured embryos in different treatment groups.

Treatment	No. of presumed zygotes	Cleavage ratio	Blastocyst rate
miR-665 mimics	482	82.16 ± 5.70^a^	42.12 ± 7.13^a^
NC mimics	477	79.45 ± 7.23^a^	33.75 ± 5.04^a*^
miR-665 Inhibitor	475	69.89 ± 6.11^b^	28.63 ± 5.87^b^
NC Inhibitor	479	79.54 ± 7.03^b*^	33.40 ± 4.90^b*^
Control	543	79.37 ± 5.56^b*^	35.17 ± 6.08^ab*^

a and b represent comparative relationships (* means *P* < 0.05) among treatments. Results are expressed as least-square mean ± SE.

Differential apoptotic staining showed that adding bta-miR-665 mimics to culture medium improved the inner cell mass (ICM) vs. total cell number (TCN) ratio 30.58% ± 2.88% (mimics vs. inhibitor), 8.55% ± 2.42% (mimics vs. Control) and reduced the number of apoptotic cells (AC) 2.53% ± 0.08% (mimics vs. inhibitor), 0.51% ± 0.01% (mimics vs. Control) (*P* < 0.05) (Table 2).

**TABLE 2:** Embryo quality assessment of Day 8 bovine embryos treated with miRNA-665 mimics or inhibitors.

Treatment	No. of blastocyst	TCN	ICM	TE	AC	ICM/TCN	AC/TCN
miR-665 mimics	16	92 ± 9.9^a^	47.6 ± 5.01^a^	44.4 ± 6.1^a^	7.86 ± .0.91^a^	51.83 ± 3.2%^a^	8.55 ± 0.3%^a^
NC mimics	20	113 ± 16.3^b^	24 ± 3.43^b^	89 ± 12.9^b^	12.52 ± 1.89^b^	21.25 ± 0.3%^b^	11.08 ± 0.2%^b^
miR-665 Inhibitor	21	129.7 ± 17.1^a^	60.8 ± 8.05^a^	68.9 ± 9.3^a^	12.45 ± 1.82^a^	46.90 ± 0.2%^a^	9.58 ± 0.2%^a^
NC Inhibitor	21	109.4 ± 13.6^b^	38.98 ± 4.79^b^	70.5 ± 9.0^b^	10.45 ± 1.57^b^	35.50 ± 0.3%^b^	9.58 ± 0.3%^b^
Control	25	119.9 ± 21^ab^	51.9 ± 9.3^ab^	67.9 ± 11.9^ab^	11.5 ± 2.24^ab^	43.31 ± 0.8%^ab^	9.60 ± 0.3%^ab^

Columns with the same sign (a, b) present significant differences (*P* < 0.05) among groups (mimics or inhibitor vs. scrambled control vs. culture media control). TE (trophectoderm), ICM (inner cell mass), AC (apoptosis cells), TCN (total cells number). Results are presented as mean ± SE.

**FIGURE 3:**
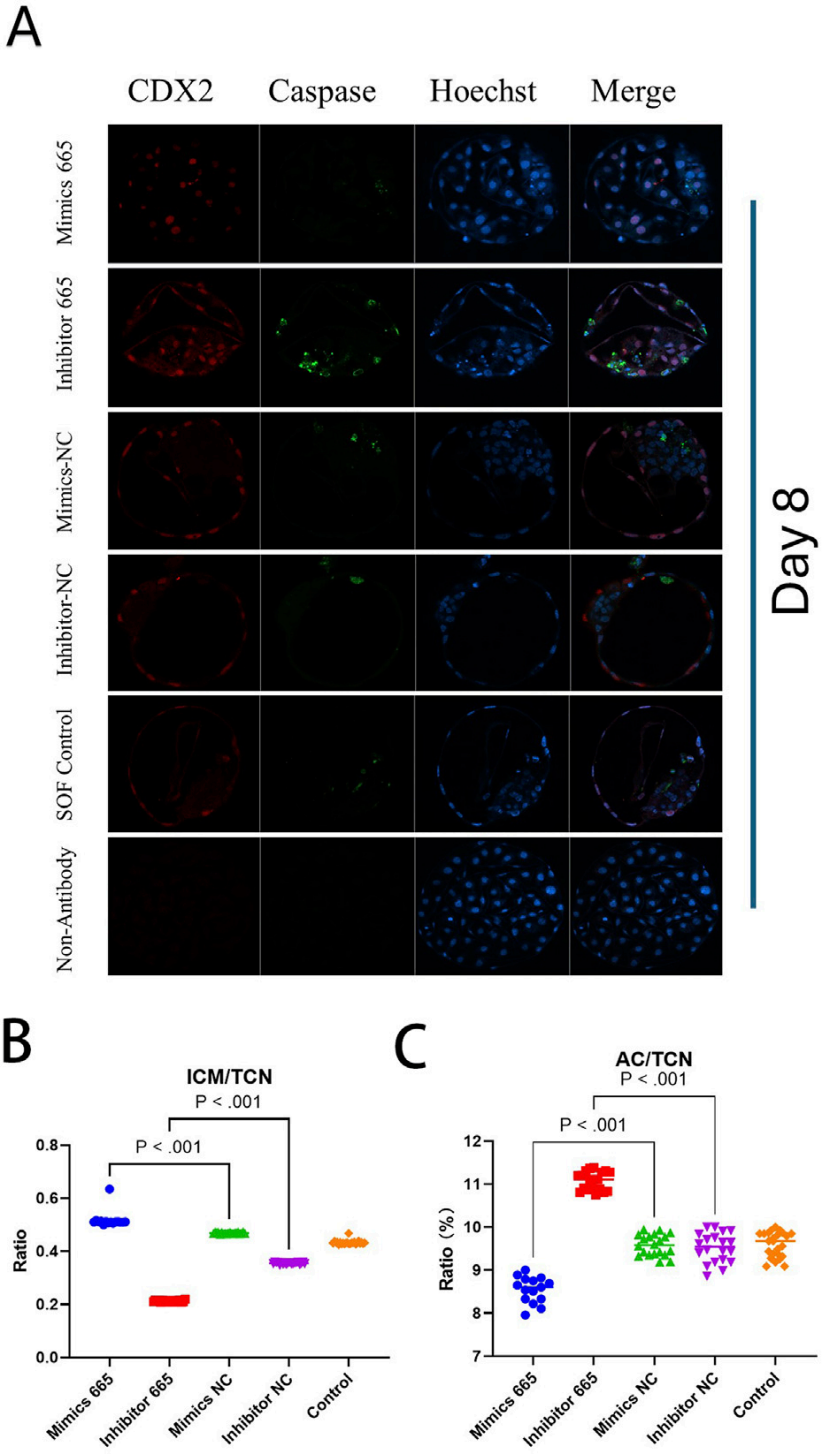
Differential staining of Day 8 blastocysts. **(A)** Differential staining using CDX2 for trophoblast cells (TE), Caspase 3 for apoptotic cells (AC) and Hoechst for total cell number (TCN). **(B)** miR-665 mimics supplemented group has higher embryonic quality (ICM/TCN rate). **(C)** mimics-665 addition group has less apoptosis cells (lower AC/TCN rate). Details are in Table 2. Results are expressed as least-square mean ± SE **(A)**.

However, the TCN in the bta-miR-665 mimics supplemented group (92 ± 9.92) was reduced compared to the mimics control group (129.75 ± 17.14) (*P* < 0.05) (Figure 3).

### Bta-miR-665 modulates the transcriptome profile of blastocysts

**FIGURE 4:**
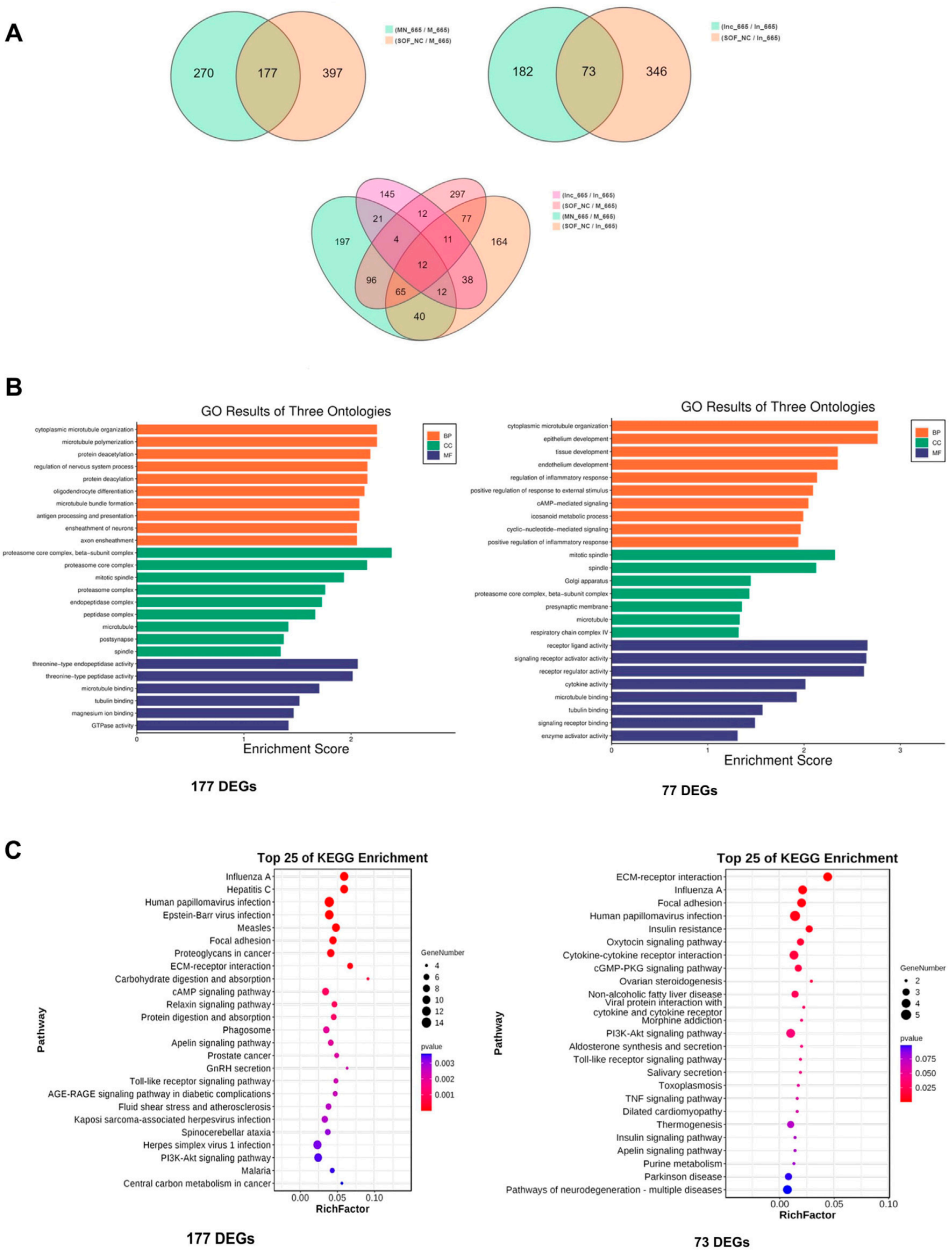
Transcriptome sequencing data analysis. **(A)** DEGs among different comparisons. **(B, C)** Top-ranking GO terms and KEGG pathways enriched in the 177 different expressed genes of mimics comparison and 73 different expressed genes of inhibitor comparison, cytoplasmic microtubule organization is the most affected biology process.

To delve deeper into the molecular mechanisms underlying the effects of bta-miR-665, we performed low-input transcriptome profiling on blastocysts from groups treated with bta-miR-665 mimics, bta-miR-665 inhibitors, and three control groups (PRJNA1099451). Comparing the bta-miR-665 mimics group vs. the NC and Control groups revealed 177 differentially expressed genes (DEGs). Comparison of the bta-miR-665 inhibitor supplemented group with the inhibitor NC and Control groups revealed 73 DEGs. Gene Ontology (GO) analysis of mimics DEGs from bta-miR-665 mimics and inhibitor supplemented groups, showed a marked association with microtubule dynamics processes (Figures 4B,C), such as “microtubule bundle” and “microtubule polymerization”. Additionally, the pathway enrichment results (Figure 4C) showed that the DEGs are associated with signaling pathways such as Regulation of Actin Cytoskeleton, PI3K-AKT signaling pathway, Focal Adhesion and Neurotransmitter-related pathways.

There are twelve common DEGs when comparing the 177 DEGs from the mimics supplemented group with the 73 DEGs from inhibitor supplemented group (Figure 4A; Supplementary Table S3). Notably, *TPPP* (Tubulin polymerization-promoting protein) and *STMN2* (Stathmin-Like 2 or Superior Cervical Ganglion-10 Protein) are genes involved in microtubule dynamics processes. Other details are shown in Supplementary Table S3.

### Bta-miR-665 downregulates AKT, STMN2 and TPPP level

**FIGURE 5:**
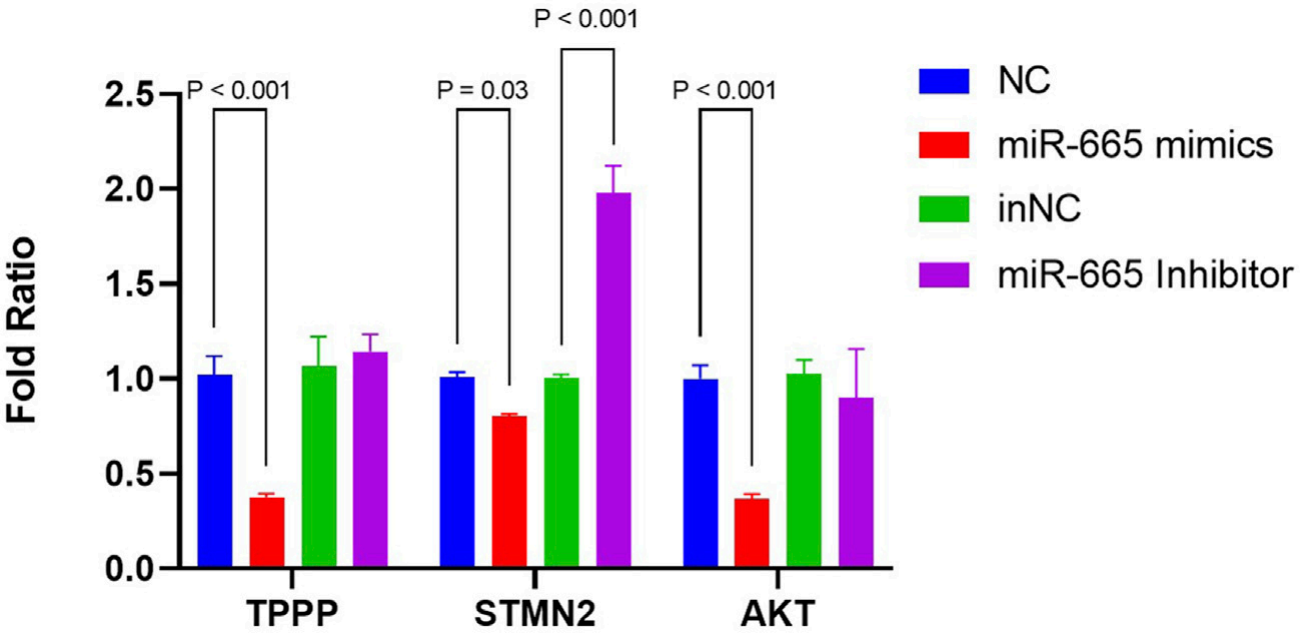
mRNA levels of TPPP, STMN2, AKT were determined by RT-qPCR. Results are presented as mean ± SE.

As microtubule dynamics was the most enriched pathway in the biology process of GO terms (Figure 4B), we further focused on *TPPP* and *STMN2*, both of which are downregulated by bta-miR-665 mimics and upregulated by bta-miR-665 inhibitors. All three of these mRNAs have potential target seed sites for bta-miR-665, as identified using the TargetScan platform. The differential apoptotic staining showed that supplementing bta-miR-665 mimics to the culture medium improved blastocyst quality. It influenced the ICM/TCN ratio (Figure 3), and since apoptosis and cell cycle/proliferation emerged from the GO pathway analysis (Figure 4C) we also included *AKT* (Protein kinase B) for further analysis. *AKT* is a well-known gene that regulates proliferation in embryos (Kalous et al., 2023; Xu et al., 2012) and the PI3K/AKT KEGG pathway showed significant enrichment in the comparison of bta-miR-665 mimics vs. NC vs. Control group, and similar in the inhibitors related comparison. RT-qPCR analysis confirmed that bta-miR-665 mimics significantly reduced *TPPP* and *AKT* mRNA levels. The *STMN2* mRNA levels were significantly downregulated by the addition of bta-miR-665 mimics and adding bta-miR-665 inhibitors did significantly upregulate *STMN2* transcription (Figure 5) (*P* < 0.05).

## Discussion

As a follow-up to a previous study where we found higher expression of bta-miR-665 in EVs in blastocyst-conditioned culture media (Pavani et al., 2022) compared to that of degenerate embryos, here we confirmed, by using RT-qPCR, that bta-miR-665 is also significantly upregulated in blastocysts proper compared to degenerated embryos. We also showed that it significantly promotes development and the quality of blastocysts by decreasing apoptotic cell counts and raising the ICM/TCN ratio and that it regulates the expression of genes involved in apoptosis and microtubule dynamics. Our results indicate that upregulation of bta-miR-665 is associated with a higher rate of embryos developing to the blastocyst stage. This makes bta-miR-665 a potentially interesting biomarker for embryonic development.

The supplementation of bta-miR-665 mimics significantly improved embryo quality and inhibition of bta-miR-665 leads to a lower zygote cleavage ratio and blastocyst ratio. The apoptotic cell count was significantly increased in the presence of bta-miR-665 inhibitors, further indicating a role in apoptotic pathways (Figure 3). This is in line with earlier findings that addition of bta-miR-665 can reduce caspase-3 levels and reduce the rate of apoptosis in luteal cells to maintain early pregnancy in ruminants (Yang et al., 2023). It was also found that bta-miR-665 shows a higher expression level in mature than immature testis from Chinese Red Steppes Cattle (Fang et al., 2021b). This may imply that bta-miR-665 in the zygote may either come from both oocytes and spermatozoa pointing to a potential role of bta-miR-665 in the degradation of certain maternal mRNAs. However, to determine whether bta-miR-665 is indeed involved in maternal mRNA clearance, more research is necessary.

Our research is the first to identify bta-miR-665 as a modulator of *TPPP*, *STMN2*, and *AKT* in embryonic development. The addition of bta-miR-665 reduces AKT levels and leads to a lower total cell number (TCN) (Figure 3A). AKT is a regulator of the cell cycle, mainly by stimulating the transition from MI to MII through activation of cyclin-dependent kinase 1 (CDK1) (Tomek and Smiljakovic, 2005). Our results show a decrease in apoptotic cells (Table 2) and downregulation of *AKT* mRNA in the group supplemented with bta-miR-665 mimics (vs. NC). It seems opposite to findings done in human cancer research where it was found that inhibition of AKT promotes apoptosis and overexpression of bta-miR-665 increases apoptosis (Dummler and Hemmings, 2007; Ashry et al., 2018; Manning and Toker, 2017; Guan et al., 2023). However, it was shown in several studies evaluating blastocyst yield that temporarily blocking meiosis resumption or reduction of PI3K/AKT activity resulted in blastocyst production equal to or even higher than that of control groups (Anas et al., 2000; de Souza et al., 2018; Pereira et al., 2020). This effect was attributed to synchronization of nuclear and cytoplasmic maturation. These findings emphasize that the regulatory effects of AKT in different pathways can lead to different results (Alcaráz et al., 2022). The fact that in our experiments bta-miR-665 mimics inhibited *AKT* mRNA but addition of bta-miR-665 inhibitor did not increase *AKT* levels may be because moderate *AKT* inhibition by bta-miR-665 may, through negative feedback regulation, activate other proliferation pathways such as *WNT*, *MAPK*, *STAT* or *mTORC* (Pópulo et al., 2012; Uko et al., 2020; Shorning et al., 2020; Kim et al., 2010) or slow down the rate of cell division contributing to normal cell cycle progression (Xu et al., 2019; Mirza et al., 2004). Moderate inhibition of AKT leads to more cellular autophagy, which will promote cellular uptake of nutrients and improve cellular quality (Dai et al., 2023). This coincides with our experimental results of having a lower TCN in bta-miR-665 mimics supplemented group compared to bta-miR-665 NC and Control group.

Our GO enrichment analysis of the DEGs identified through transcriptomic analysis showed that the effects of bta-miR-665 on embryonic development are mainly situated in processes related to microtubule synthesis and assembly (Figures 4B,C) (Finkielstein et al., 2001; Ulrich et al., 2016). Stathmins (STMNs) are well-established regulators of microtubule dynamics during mitotic spindle formation, influencing the development of mouse and bovine trophoblasts (Schulz et al., 2009; Hosseini et al., 2015). *STMN2* has been shown to interact with several other proteins that are involved in cellular processes such as promoting cytoskeletal assembly and apoptosis (Pellier-Monnin et al., 2001; Tortoriello et al., 2014; Bahn et al., 2002). The reduction of *STMN2* levels affects the timing or progression of cell division, leading to more efficient DNA repair (Klim et al., 2019). This conforms with our results that supplementation of bta-miR-665 mimics to the embryo culture media leads to the downregulation of *STMN2* mRNA (Figure 5) and subsequently to a higher blastocyst rate and quality (high ICM/TCN ratio) and lower apoptotic cell ratio (Figure 3).

The dynamic equilibrium maintained in the microtubule network has a profound effect on the development of the embryo and the functioning of its cells (Job et al., 2003). Because TPPP serves as a microtubule nucleation factor, it is one of the essential regulators of this equilibrium despite having little impact on the pace at which microtubules develop (Fu et al., 2019). It has been shown that TPPP protein extracted from bovine brains, when injected into *Drosophila* embryos, has a significant effect on spindle formation and nuclear membrane rupture processes, which play a decisive role in embryonic development, especially during cell division (Tirián et al., 2003). In another study of *Drosophila* embryos, overexpression of TPPP led to abnormal proliferation of the microtubule network and this excessive microtubule polymerisation interfered with normal cell division, leading to improper chromosome alignment and segregation, which in turn triggered chromosomal abnormalities and led to poor embryonic development (Vicente and Wordeman, 2019). These findings highlight the importance of TPPP in maintaining the equilibrium of the microtubule network, as well as during accurate cell division. As we have shown that addition of bta-miR-665 lowers TPPP mRNA levels (Figure 5), it can be expected that bta-miR-665 is involved in these processes. It was also shown that during *Drosophila* and human embryonic development, the development of axons and secretory vesicles is affected by TPPP and in mouse embryo implantation experiments, the disassembly of microtubules leads to implantation failure (Mino et al., 2016; Shukla et al., 2019; Kanaumi et al., 2013). Therefore, the inhibition of TPPP by bta-miR-665 may not only help to avoid improper chromosome alignment and segregation, but also promote the stability of cellular morphology and proper intracellular substance transport by regulating the dynamic balance of microtubules. A link with AKT may be made in this respect. As AKT has been shown to control synaptic strength, AKT inhibition may slow the synthesis of nerve synapse-associated proteins and promote stem cell differentiation in the brain. This finding supports the notion that *AKT* can also regulate microtubule development (Wang et al., 2003). We hypothesise that during mitosis in early embryos, well-developed cells will appropriately inhibit excessive microtubule nucleation processes to reduce the rate of cell division.

Based on our current findings, we suggest that bta-miR-665 can be a potential biomarker for embryo development and quality. However, future studies in a clinical setting, focusing on the analysis of miRNA expression profiles of bta-miR-665 from embryo spent media, are necessary to validate its utility as a biomarker. In conclusion, bta-miR-665 has a positive effect on embryo development through its influence on microtubule dynamics in conjunction with apoptosis.

## Data Availability

The datasets presented in this study can be found in online repositories. The names of the repository/repositories and accession number(s) can be found in the article/Supplementary Material.
