# Supplementary material for: Bta-miR-665 improves bovine blastocyst development through its influence on microtubule dynamics and apoptosis
Source: Front Genet. 2024 Oct 16;15:1437695. doi: 10.3389/fgene.2024.1437695 (PMC11521815; doi:10.3389/fgene.2024.1437695)
Supplement: Supplementary file 5 [file DataSheet1.docx]

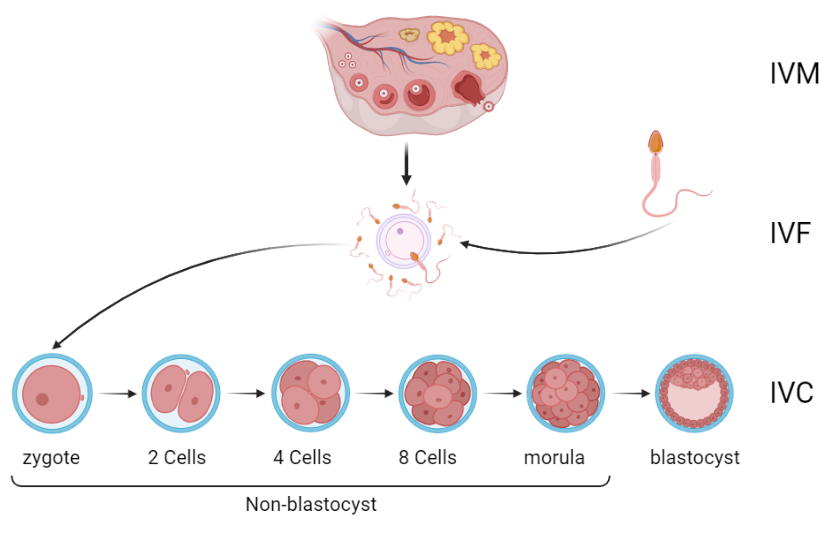


**Supplementary Figure 1: Embryo development process.** We set the embryos between Zygotes to morula states as the non-blastocyst group (degenerated).


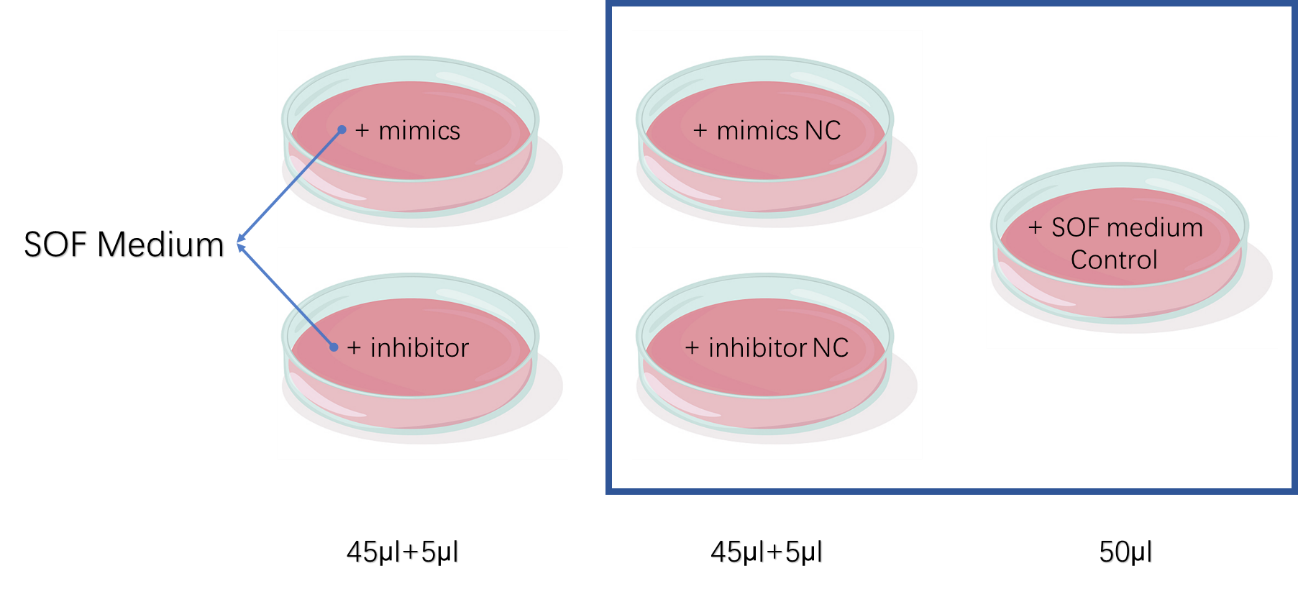


**Supplementary Figure 2: Supplements addition and groups settings.** Add total 50μl to all treatment groups, and set up experimental groups and negative control (NC) groups


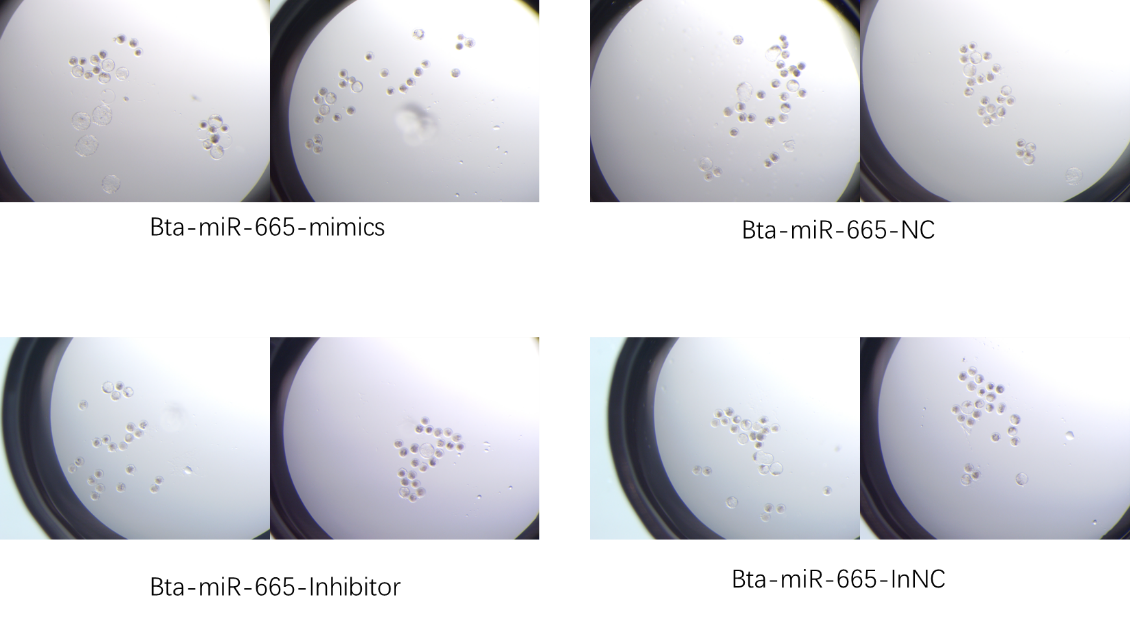


**Supplementary Figure 3: The growth status of embryos in different supplement groups at day 8.**

CDX2 Caspase 3 Hoechest Merge


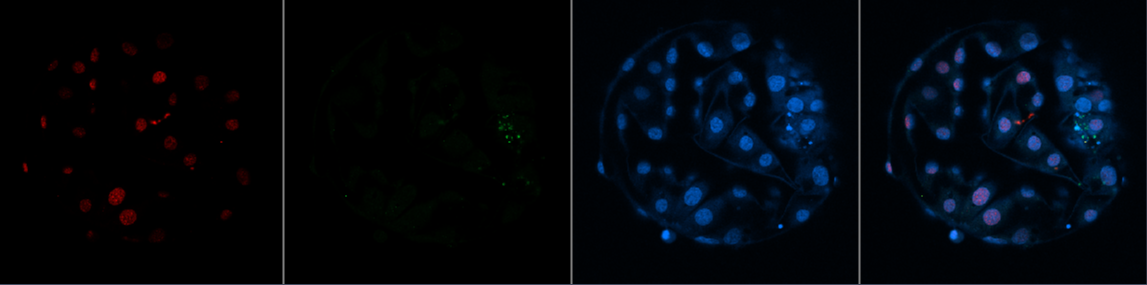


Bta-miR-665 mimics


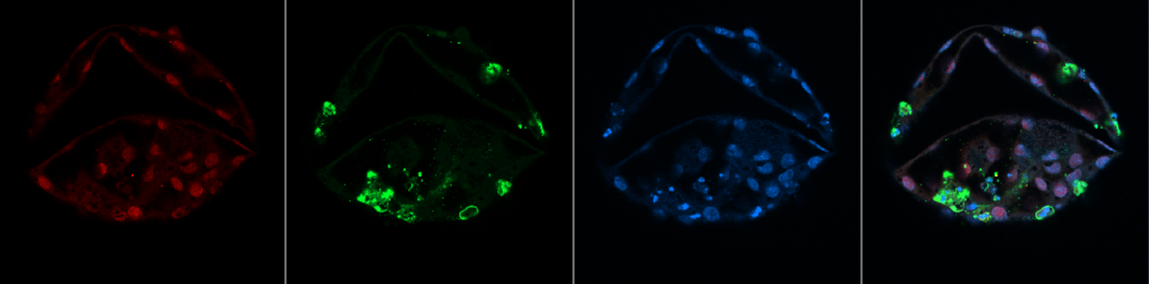


Bta-miR-665 inhibitor


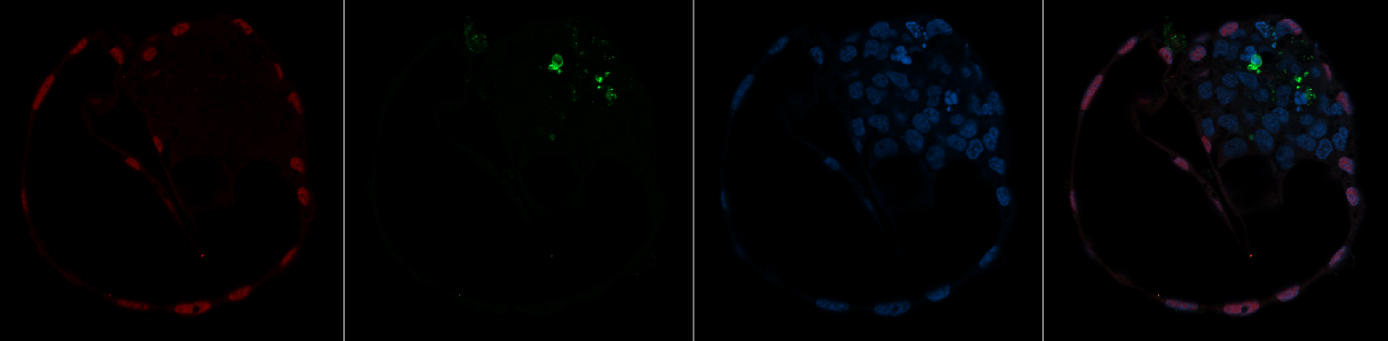


Mimics Negative Control (NC)


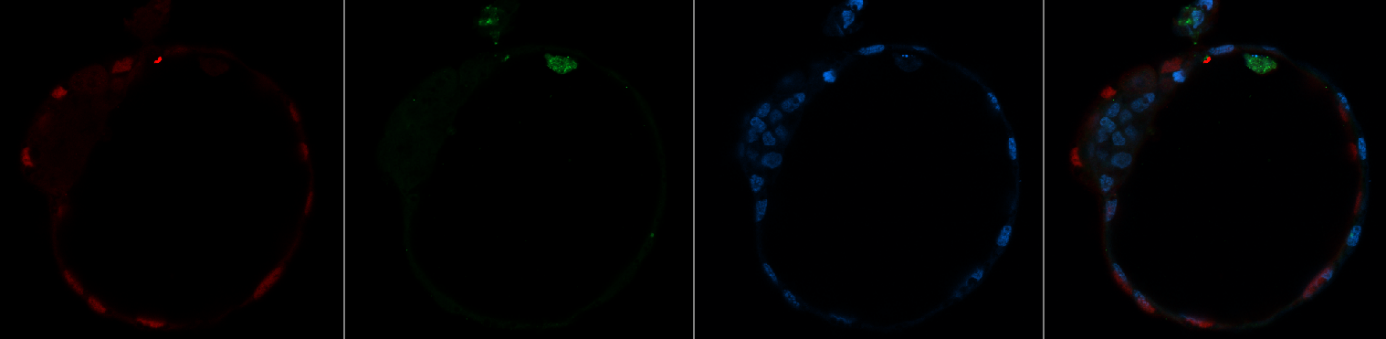


Inhibitor Negative Control (inNC)


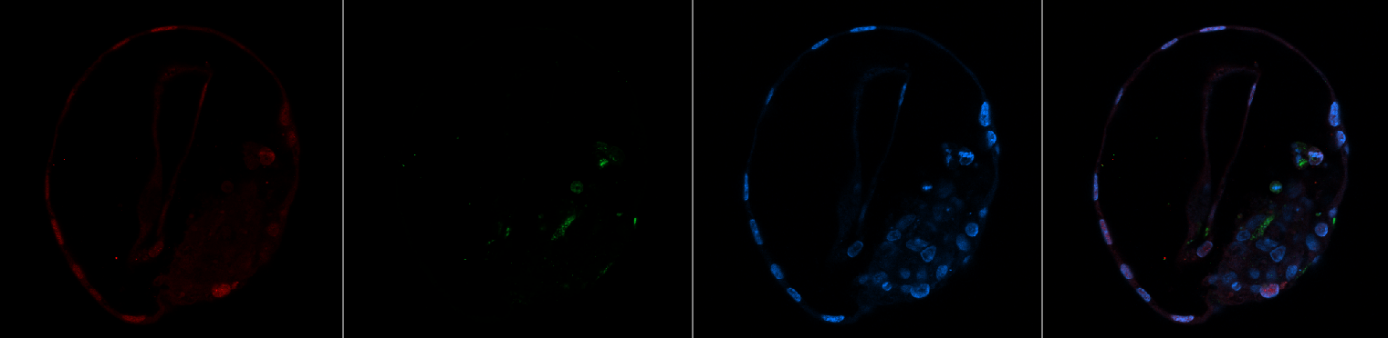


SOF media Control (Control)


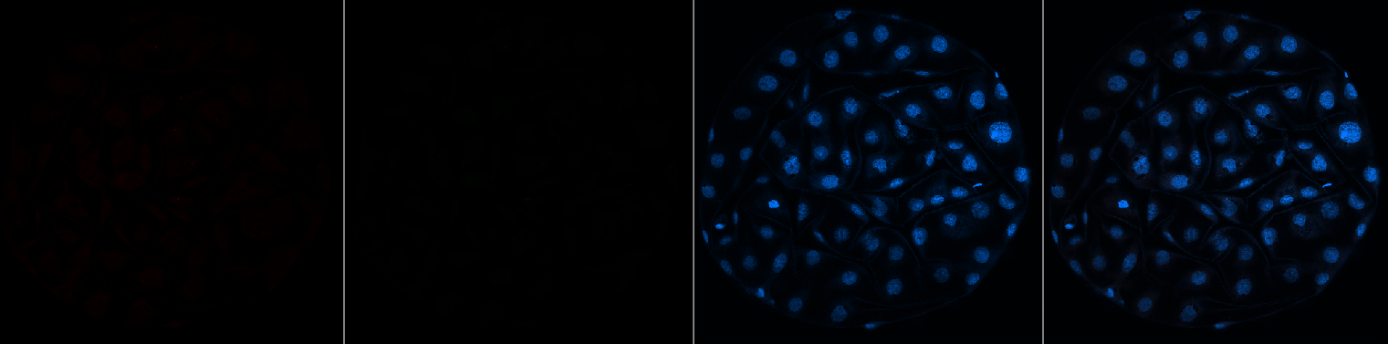


No antibody control

**Supplementary Figure 4: Differential Staining of blastocysts.** Differential staining using CDX2 for trophoblast cells (TE), Caspase 3 for apoptotic cells (AC) and Hoechst for total cell number (TCN)


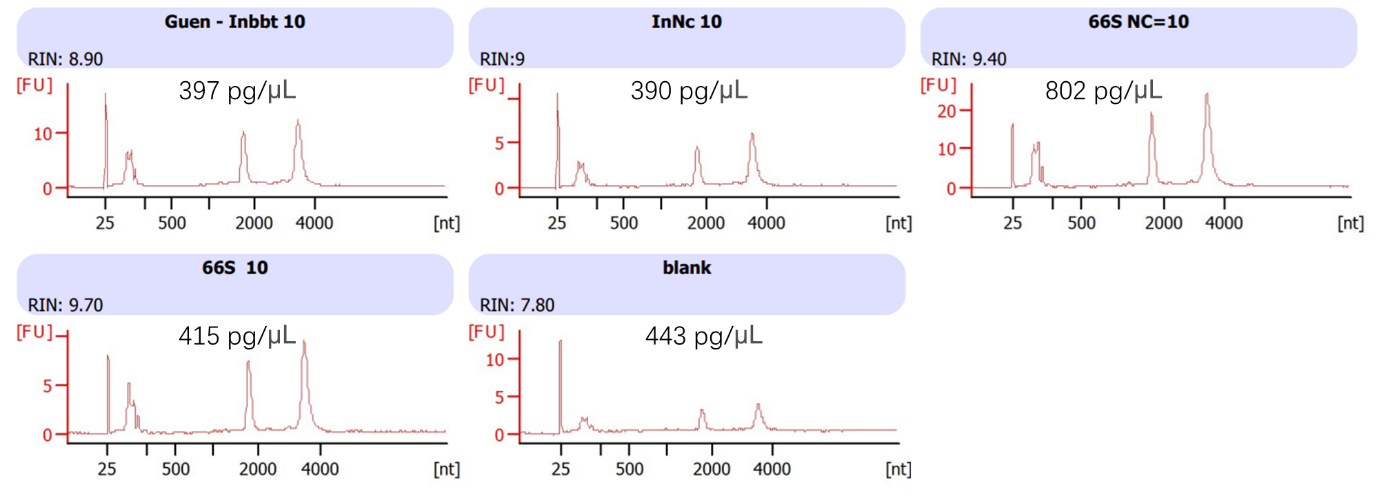


**Supplementary Figure 5: Bioanalyzer results of the quality of total-RNA extracted from blastocysts of different treatments.**

**Supplementary Table 1: Primers used in this study.**

| Target | Sequence | Annealing Tm(℃) |
| --- | --- | --- |
| Bta-miR-665 | ACCAGTAGGCCGAGGCCCCT | 60 |
| STMN2 Forward | CCTGATCTGCTCTTGCTTCTAC | 58 |
| STMN2 Reverse | GAGGCACGCTTGTTGATTTG | 58 |
| TPPP Forward | CCCTGAACTCACCTTTCCTAAC | 58 |
| TPPP Reverse | CTGGTTCTGGGAGACACTTTG | 58 |
| AKT Forward | GCGTGACCATGAATGAGTTTG | 62 |
| AKT Reverse | CCACGATGACCTCCTTCTTTAG | 62 |

**Supplementary Table 2: Abbreviation**

| Abbreviation | Full Name |
| --- | --- |
| Bta-miR-665 | Bovine MicroRNA 665 |
| PBS | Phosphate buffered saline |
| RNAi | RNA interference |
| AKT | Protein kinase B |
| STMN2 | Stathmin-Like 2/Superior Cervical Ganglion-10 Protein |
| TPPP | Tubulin polymerization-promoting protein |

**Supplementary Table 3: 12 DEGs identified from both 177 and 73 DEGs**

| Gene ID | Gene Symbol | log2 (MN_665 / M_665) | FDR (MN_665 / M_665) | log2 (SOF_NC / M_665) | FDR (SOF_NC / M_665) | log2 (Inc_665 / In_665) | FDR (Inc_665 / In_665) | log2 (SOF_NC / In_665) | FDR (SOF_NC / In_665) |
| --- | --- | --- | --- | --- | --- | --- | --- | --- | --- |
| 768081 | TNFSF18 | -2.87 | 2.50E-16 | -3.25 | 3.10E-19 | -3.24 | 1.40E-19 | -3.38 | 1.10E-19 |
| 497204 | UBA7 | -1.42 | 3.00E-09 | -1.14 | 1.00E-07 | -2.62 | 1.80E-51 | -2.59 | 2.70E-48 |
| 510377 | SP140 | -1.17 | 5.19E-04 | -1.52 | 7.22E-06 | -2.32 | 2.00E-19 | -2.72 | 3.90E-22 |
| 282013 | PSMB8 | -1.1 | 1.15E-05 | -1.48 | 6.00E-09 | -2.41 | 2.90E-19 | -1.9 | 8.50E-14 |
| 508394 | CASQ1 | -1.02 | 2.92E-04 | -1.17 | 1.61E-05 | -3.02 | 2.50E-14 | -1.13 | 2.63E-04 |
| 521764 | MYH7B | 1.17 | 1.50E-32 | 1.91 | 3.40E-68 | -3.02 | 1.02E-106 | -1.96 | 3.20E-61 |
| 100139352 | DENND6B | 1.3 | 7.80E-28 | 2.28 | 2.30E-61 | -1.15 | 8.70E-11 | -1.21 | 1.00E-10 |
| 34991 | STMN2 | 1.71 | 7.00E-03 | 3.25 | 1.20E-10 | -0.85 | 4.97E-04 | -0.52 | 6.15E-05 |
| 509821 | ELMO1 | 1.9 | 9.30E-24 | 1.74 | 5.20E-23 | -3.26 | 2.50E-58 | -2.22 | 1.10E-36 |
| 280968 | TPPP | 2.16 | 2.11E-05 | 1.81 | 2.95E-04 | -1.53 | 1.90E-08 | -4.36 | 1.11E-05 |
| 787080 | IGSF22 | 2.77 | 6.20E-32 | 3.07 | 1.10E-41 | -2.29 | 1.50E-18 | -3.07 | 9.70E-48 |
| 521567 | TRIM64 | 3.27 | 1.40E-14 | 3.29 | 8.50E-14 | -2.38 | 1.61E-06 | -3.13 | 1.90E-15 |
